# Compliance with ecological momentary assessment protocols in substance users: a meta‐analysis

**DOI:** 10.1111/add.14503

**Published:** 2018-12-21

**Authors:** Andrew Jones, Danielle Remmerswaal, Ilse Verveer, Eric Robinson, Ingmar H. A. Franken, Cheng K. Fred Wen, Matt Field

**Affiliations:** ^1^ Psychological Sciences University of Liverpool UK; ^2^ UK Centre for Tobacco and Alcohol Studies (UKCTAS), Liverpool UK; ^3^ Department of Psychology, Education and Child Studies Erasmus University Rotterdam the Netherlands; ^4^ Center for Self‐Report Science University of Southern California Los Angeles CA USA

**Keywords:** Addiction, compliance, dependence, ecological momentary assessment, meta‐analysis, substance use

## Abstract

**Background and Aims:**

While there are considerable benefits to Ecological Momentary Assessment (EMA), poor compliance with assessment protocols has been identified as a limitation, particularly in substance users. Our aim was to identify the pooled compliance rate of EMA studies in substance users and examine variables that may influence compliance with EMA protocols, such as the length and frequency of assessments.

**Design:**

A meta‐analysis and meta‐regression of all possible studies (randomized controlled trials and longitudinal) which incorporated EMA protocols, examining substance use.

**Setting:**

Studies took place from 1998 to 2017, in numerous countries world‐wide.

**Participants:**

One hundred and twenty‐six studies were identified, contributing a total of 19 431 participants (52.32% male, mean age = 28.86).

**Measurements:**

Compliance data, the proportion of responses to the study protocol, were extracted from each study alongside prompt frequency, total length of assessment period, substance use population and device used to administer EMA prompts.

**Findings:**

The pooled compliance rate across all studies was 75.06% [95% confidence interval (CI) = 72.37%, 77.65%]. There was no evidence that compliance rates were significantly associated with prompt frequency [*Q*(3) = 7.35, *P* = 0.061], length of assessment period [*Q*(2) = 2.40, *P* = 0.301], substance type [*Q*(3) = 6.30, *P* = 0.098] or device administration [*Q*(4) = 4.28, *P* = 0.369]. However, dependent samples (69.80%) had lower compliance rates than non‐dependent samples [76.02%; *Q*(1) = 4.13, *P* = 0.042].

**Conclusions:**

The pooled compliance rate for Ecological Momentary Assessment studies in substance‐using populations from 1998 to 2017 was lower than the recommended rate of 80%, and was not associated with frequency or duration of assessments.

## Introduction

Ecological Momentary Assessment (EMA) refers to a variety of research techniques that allow for ‘in the moment’ data capture, which often takes place in naturalistic rather than standard laboratory settings 1. EMA methods provide patterns of rich data which can model relationships between variables over time in a way that cross‐sectional assessment cannot. The popularity of EMA methods has increased rapidly in recent years 2, owing in part to the development of mobile technology and software packages that enable sophisticated data analyses 3, 4. A typical EMA study may send participants a number of signalled prompts on a portable device. When these prompts are received, participants are required to recall their current thoughts, behaviours and feelings; more recent studies have introduced cognitive and behavioural tasks 5, 6. These prompts may occur randomly throughout the course of the day or during a fixed epoch (known as signal‐contingent responding). Participants may also be asked to initiate assessments in response to specific events (known as event‐contingent responding), such as awareness of intrusive cravings, temptations to use drugs 6 or soon after having smoked a cigarette 7.

EMA has numerous benefits over laboratory‐based assessments, particularly when investigating substance use 8. Substance use is a discrete episodic behaviour, influenced by immediate environmental factors (e.g. substance‐related cues 9) and internal factors such as stress 10, 11 and craving 12, 13, which can be difficult (both practically and ethically) to model in the laboratory. Furthermore, the assessment of substance use and its correlates in the laboratory often relies on retrospective recall which can be biased, particularly when measured following periods of drug/alcohol intoxication. For example, Monk *et al*. 14 demonstrated that a greater number of alcoholic drinks were reported in real time using EMA than when assessed retrospectively (see also 15). Similarly, individuals’ substance use may be suppressed in contexts that are not typically associated with substance use; for example, Monk *et al*. 16 demonstrated increased alcohol consumption during an experiment conducted in a pub compared to a library. In relation to this, participants’ expectations of drug effects may be suppressed in the laboratory, with self‐reported alcohol outcome expectancies differing between typical drinking (pub) and atypical contexts (laboratory 17, 18). Finally, reliable self‐report assessment of illegal drug use can be difficult to obtain due to biases in retrospective recall 19, 20. However, EMA designs allow for repeated, proximal assessments which improve accuracy 21. While EMA has various methodological advantages over laboratory‐based research it can also present significant challenges. Outside the laboratory the experimenter relinquishes their control of variables which might be of interest 22. There is also the complexity of everyday life (e.g. distractions) and competing priorities, which may negatively impact participant compliance and increase missing data. For example, participants may be unable or unwilling to complete assessments when at work, studying or when out with friends 23. These issues may be magnified in substance users who have ‘unstable’ life‐styles 24.

Missing data through non‐compliance can have a significant effect on statistical power, but also conclusions that can be drawn through statistical inference 25, particularly when data are missing systematically (i.e. not at random), because this introduced bias. For example, participants may regularly miss specific assessments (e.g. if they are working during the hours that a prompt is due), rather than randomly missing a prompt due to being in an unscheduled meeting or driving. Researchers have suggested that the majority of non‐compliance in EMA research is systematic 26. Therefore, the challenge is to design EMA testing protocols that facilitate high rates of compliance. However, it is entirely possible that experimental protocols for EMA studies might contribute to systematic bias in missing data through non‐compliance. There are many theoretical and practical decisions an experimenter needs to make when designing an EMA study, such as the quantity and frequency of prompts, length of assessment periods and whether to reimburse participants for compliance, etc. 27. Despite their importance, few individual studies have directly examined the effect of these procedural variables on compliance.

Sokolovsky *et al*. 28 examined factors in a single study that predicted poor compliance to an EMA protocol among adolescent smokers. They demonstrated that longer inter‐prompt intervals decreased compliance, which the authors hypothesized was due to participant disengagement. Furthermore, numerous studies have examined compliance as a function of total assessment period (i.e. duration of study), and demonstrated that compliance declines as the study progresses 29, 30, 31. However, others have failed to replicate these observations 7 32. One study of alcohol consumption demonstrated that morning assessments (between 8 a.m. and 11 a.m.) had poorer compliance 33 than assessments prompted later in the day, and finally compliance is often improved through financial incentives 34, 35.

Alongside procedural variables, individual differences may also influence non‐compliance. Messiah *et al*. 36 demonstrated that males, polysubstance users and those with a diagnosis of substance use disorder during the previous 12 months were less likely to respond to EMA prompts. Turner *et al*. 37 examined factors which influenced discontinuation in responding to daily EMA prompts in substance‐using men who have sex with men, and demonstrated better compliance in younger participants and those who were educated to college‐level. Smokers who relapsed (versus those who did not) following a quit attempt also had higher rates of non‐compliance 38, as did alcoholics 24. Finally, one study with problem drinkers reported the perhaps counterintuitive finding that individual differences in social desirability were unrelated to compliance in problem drinkers 39, although this issue has not been comprehensively investigated.

Despite its importance, there has been no comprehensive and systematic investigation of compliance rates in the substance use EMA literature. In a narrative review, Shiffman *et al*. 8 reported compliance rates ranging from 50 to 90%, but stated: ‘it is unclear what caused this substantial variation’. In a related field, Liao *et al*. 40 reported an average compliance rate of 71.3% (range 43.8–95.9%) in 13 studies of diet and physical activity; however, many of these studies utilized paper‐and‐pencil diary methods, where the veracity of compliance could not be tested. A recent meta‐analysis examining compliance in EMA studies assessing behaviours, subjective experiences and contextual information in children and adolescents reported an overall compliance rate of 78.3% 41, with no difference between clinical and non‐clinical samples. However, when analysing the samples separately there was a decrease in compliance, with increased frequency of daily responding in non‐samples, but this relationship was reversed in clinical samples.

Although there is no reported ‘gold standard’ of compliance, several studies recommend compliance rates of at least 80% 26, 37, 42. 1To our knowledge. there is no clear justification in the literature for 80% as a benchmark for recommended compliance although it is, in our opinion, appropriate, because although we would expect participants to be compliant the majority of the time, it is not practical or realistic to expect close to perfect compliance. Therefore, the current meta‐analysis had two primary aims: (1) we aimed to obtain a comprehensive pooled estimate of compliance rates taken from substance‐using samples and compare this to the recommendation of 80%; and (2) we aimed to test the role of our identified moderators that may influence compliance in EMA protocols, including prompt frequency, total assessment duration, assessment length, clinical diagnosis, type of substance and device administration. Investigation of these moderators were included a priori, as (i) individual studies have reported a relationship with compliance and (ii) the majority of studies included this procedural information. We also conducted further exploratory moderation analyses on whether participants were trained in the protocol, the type of reimbursement offered and the presence of event‐contingent assessments.

## Methods

### Information sources and search strategy

We searched three comprehensive academic literature databases (Pubmed, PsycInfo and Scopus) from inception to December 2017. The search terms included terms related to EMA (ecological momentary* OR experience sampling OR event sampling OR daily diary) and substance use (Addiction OR substance use OR dependence OR drugs OR alcohol OR cannabi* or marijuana OR smoking OR tobacco OR cigarette OR cocaine OR heroin OR ecstasy OR MDMA). Results were limited to articles written in English. The search strategy and eligibility criteria, analyses plan and hypotheses were pre‐registered on Open Science Framework prior to commencement of the searches (https://osf.io/thmgy/). The completed PRISMA (Preferred Reporting Items for Systematic Reviews and Meta‐Analyses) checklist for this study can be found in the Supporting information, Table S1).

### Eligibility criteria

In order to be included in the meta‐analyses, articles were required to (i) employ EMA methodologies (i.e. not repeated laboratory‐based assessments), (ii) utilize mobile technologies for EMA data collection that did not require face‐to‐face contact with researchers (PDAs, smartphones, internet), (iii) assess the use of substances of abuse (alcohol, nicotine, cannabis, etc.) either as a study outcome or predictor of a study outcome and (iv) include ‘signal‐contingent’ assessments.

Studies were excluded if (i) data were collected in the presence of an experimenter in the laboratory, (ii) data were collected less than once per day (to ensure consistency with previous analyses), (iii) only ‘event‐contingent’ assessments were used or (iv) EMA was implemented using paper‐and‐pencil diary methods. We excluded EMA studies which only examined event‐contingent assessments, as it is not possible to report compliance to self‐initiated reports 3. Paper‐and‐pencil methods were excluded, as they are subject to bias through backfilling or hoarding, and time/date of completed entries cannot be independently verified 24, 43. Studies were also excluded if the (re)analysis of the same data or a subset of these data occurred (for example, numerous publications have arisen from the data set reported in 44 to ensure that all data in the meta‐analysis were independent. The majority of studies contributed one effect size for all participants recruited. Three studies provided compliance rates separately for subgroups (e.g. males and females) 45, 46, 47. We used the mean compliance rate across subgroups adjusted for sample size in these cases.

### Extraction and coding

The outcome variable (‘compliance’) was the percentage of signal contingent assessments that were responded to in line with the experimental protocol. The majority of the studies included these data. Where studies did not include these data, but included the number of daily assessments, length of assessment period, number of participants and total number of completed assessments, we were able to calculate the mean compliance rate throughout the whole sample. We also extracted the following variables to examine our a priori hypotheses: the number of prompts (signals) per day, total length of assessment period, duration of individual assessments, type of substance use (alcohol, tobacco, marijuana, opioids/cocaine, mixed), whether assessments were prompted (e.g. a text signalling a prompt needed to be completed within a given time‐frame) or scheduled during fixed time intervals (e.g. 2 p.m. to 7 p.m.), whether financial or other incentives were offered for completion of the study or in proportion to compliance and whether the sample had a clinical diagnosis of substance use. For exploratory analyses (not originally pre‐registered) we extracted whether participants were also requested to make event‐contingent assessments (coded as yes or no), whether articles explicitly stated if training for the EMA protocol was provided to participants (coded as yes or not reported), whether participants were receiving/had received treatment for their substance use (present, absent), whether participants had self‐reported motivation to quit/cut down (present, absent) and year of publication. We conducted moderator analyses on each of these variables separately. Finally, we also extracted the proportion of individuals in each study who were excluded due to poor compliance (see Supporting information). There was high agreement in the coding of extracted data (> 97%), and any disagreements were resolved through discussion.

We examined our confirmatory hypotheses, using the same moderator coding as Wen *et al*. 41 and as outlined in our pre‐registration. Specifically, we grouped number of prompt frequency (number of prompts per day) as one prompt, two to three prompts, four to five prompts and six or more prompts. Some studies provided a range of prompts per day (e.g. five to seven); in these cases we took the average number of prompts if reported (e.g. five, six) and if not we took the median number (e.g. six). We coded total length of assessment as ≤ 1 week, > 1 week but ≤ 2 weeks and > 2 weeks using categories attenuated for non‐normally distributed data, when clustered around common time‐frames for studies (e.g. 1 week). We also used the study‐level data (e.g. exact prompt frequency and total length of assessment) and included these as covariates in a separate meta‐regression model (Supporting information). A number of effect sizes (*k* = 43; 34.1%) had corresponding information on individual assessment duration (in minutes), therefore we analysed this in isolation in order to preserve statistical power. If a range was given (e.g. 5–10 minutes, see 48) we used the middle value (7.5 minutes in this case). For purposes of administration, we separated devices into personal digital assistants (PDAs), smartphone‐owned, smartphone‐loaned, internet‐based or interactive voice responding. Some studies loaned participants a smartphone, regardless of whether or not they owned their own, and as such were coded as smartphone loaned. Other studies (e.g. 49) allowed participants to use their own phone or loan a phone if they did not own one; we coded these as ‘smartphone‐owned’ due to the large number of individuals who now own their own smartphones (however, no individual‐level data on number of loaned phones were presented in these studies). Interactive voice responding requires individuals to call a number and answer questions, and as such the EMA protocol is not administered directly on a device. To code whether participants were trained on the EMA protocol we examined whether there was any mention of training or familiarization sessions (e.g. ‘research assistants… provided a brief training on how to download and navigate the mobile app’ 50, p. 1249).

### Statistical analyses

Random‐effects restricted maximum likelihood estimator meta‐analyses were conducted. Random‐effects models were used due to considerable heterogeneity, and we used restricted maximum likelihood as they are more conservative than maximum likelihood models 51 and perform better than other random‐effects models 52. To compute effect sizes we transformed proportion of completed assessments by the square root of their arcsine. Square‐root arcsine transformations were used rather than raw proportions to reduce variance instability and preclude confidence intervals falling outside the range of 0.0–1.0 53. The transformed compliance was normally distributed [skewness statistic −0.329, standard error (SE) = 0.216]. We estimated the SE using the equation **√**{(1/np) + [1/n(1–p)]}, where ‘n’ was the sample size and ‘p’ was the proportion. As compliance rates are clustered within participants we adjusted the sample size by the within‐ and between‐study variance (intraclass correlation coefficient), similar to the method described by Wen *et al*. 41). Twenty‐two (17.2%) compliance rates had accompanying information for individual variation [standard deviation (SD)]. We took the median of the available SDs and imputed this for all studies that did not report this, and we also conducted sensitivity analyses by computing the SEs using the 25th and 75th percentiles of available SDs. To present interpretable data, we inverse logit‐transformed the proportions and then converted these back to a percentage following our analyses. The *I* ^2^ statistic was used to quantify heterogeneity across the effect sizes, and the *Q* statistic was included as a test of heterogeneity reduction through the inclusion of moderators. All analyses were conducted in JASP (2017), which implements the metafor package from R 54.

Some studies separated samples by substance use, gender or pre–post intervention; these cases contributed a single effect size 45, 46, 47. No outliers were detected using our pre‐registered methods of examining extreme values in the distribution (*Z* > 3.30) or effect sizes with confidence intervals (CIs) which did not overlap any other individual CIs or the pooled estimate. Four cases were identified by Cook's Distance to be influential 30, 55, 56, 57, therefore we analysed all data with these data points included and excluded. Data are reported with the data points included unless exclusion significantly influenced the results.

## Results

### Articles identified

**Figure 1:**
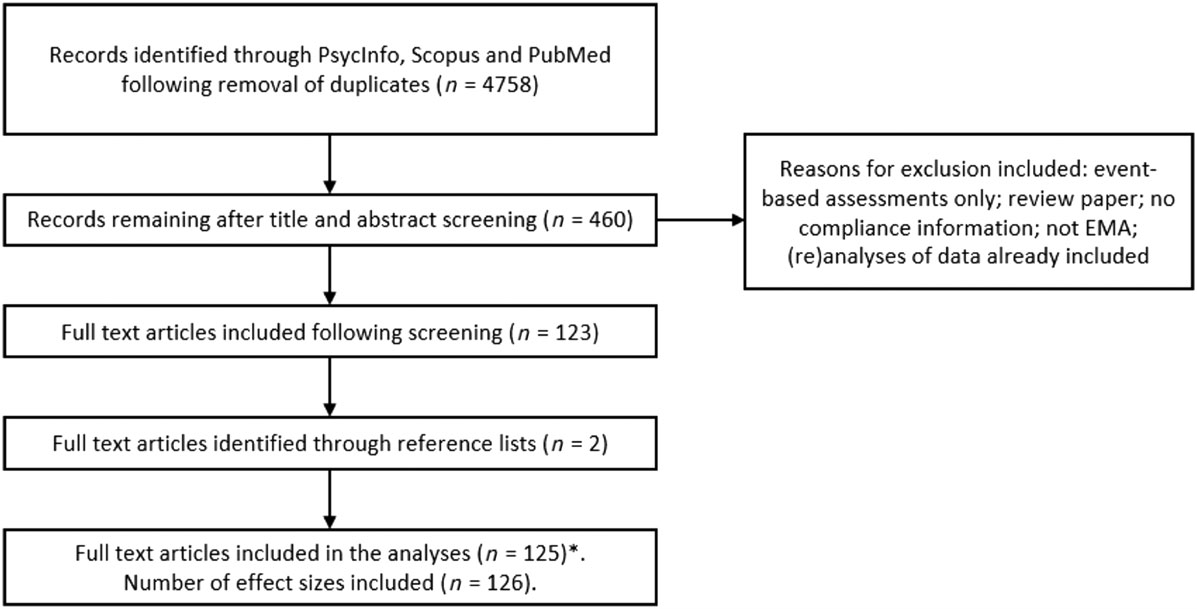
Flow‐chart of the search procedure and studies included in the analyses. *One article 70 contained two ecological momentary assessment (EMA) studies

The initial searches included 4758 articles after removal of duplicates. Title and abstracts were screened and cross‐checked by A.J., D.R. and I.V., and 4298 were removed. The remaining articles were full‐text screened, and after removal of those that did not meet our eligibility criteria, 125 articles published between 1998 to 2017 remained reporting data on a total of 19 431 individuals (see Fig. 1 for more information and Open Science Framework for list of articles).

### Study characteristics

Distributions of participant demographic and moderator variables are summarized in Table 1. There were negative associations between individual assessment duration and prompt frequency (*r* = − 0.52, *P* < 0.001) and length of assessment period and prompt frequency (*r* = − 0.18, *P* = 0.035). There was no significant association between length of assessment period and individual assessment duration (*r* = 0.11, *P* = 0.49). The large SD in length of assessment period was due to eight outliers which had assessment periods greater than 84 days; removal of these outliers did not substantially influence the results reported below.

**Table 1:** Participant demographic and moderator variable summaries.

	Mean	SD	Range
Age	28.86	10.21	12.50–52.00
Gender (% male)	52.32	22.61%	0–100
Number of participants	154.21	214.80	10–1054
Number of daily prompts	3.63	2.03	1–9
Length of assessment period (days)^a^	30.29	68.51	3–730
Length of individual assessment (mins)	3.94	2.40	1–10
	Total		
Number of studies	126		

a Mean = 18.89, standard deviation (SD) = 11.52, range = 3–60, after removal of eight outliers.

Neither participant age (coefficient = < 0.00, SE = < 0.01, *Z* = 0.15, 95% CI < −0.01; > 0.01, *P* = 0.885) nor gender distribution (coefficient = < −0.00, SE = < 0.01, Z = −0.44, 95% CI < −0.01; > 0.01, *P* = 0.659) were reliably related to compliance rates.

### Overall compliance rate

**Figure 2:**
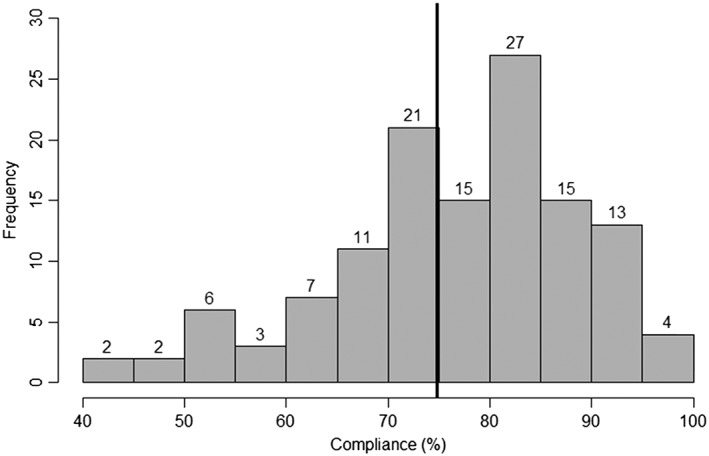
Distribution of compliance rates across studies in meta‐analyses/regression (solid black vertical line represents pooled compliance rate of 75.06%)

Across *k* = 126 effect sizes the overall compliance rate was 75.06% (95% CI = 72.37%, 77.65%). The confidence intervals did not include 80%, suggesting that overall compliance was significantly different from 80% (*P* < 0.05). There was considerable heterogeneity among compliance rates (*I* ^2^ *=* 44.76%). Removal of the influential cases did not substantially influence the pooled compliance rate or CIs (75.70%; 95% CI = 73.49%, 77.84%), but reduced heterogeneity estimates (*I* ^2^ *=* 16.04). One hundred and thirteen compliance rates (89.7%) had an upper‐bound CI which overlapped 80%, suggesting that compliance was adequate in these cases. The distribution of study‐level compliance rates can be seen in Fig. 2. Our sensitivity analyses (using estimated SDs, data not shown) did not substantially influence compliance estimates.

### Pre‐registered analyses

#### Prompt frequency, total length of assessment and individual assessment duration (see table 2)

**Table 2:** Compliance rates (95% CIs) for pre‐registered and exploratory analyses.

Pre‐registered analyses	n	Pooled compliance (95% CI)	Q (d.f.)	P	
Overall compliance rate	126	75.06 (72.37, 77.65)			
Prompt frequency			7.35 (3)	0.061	
1 per day	32	76.77 (70.43–82.64)			
2–3 per day	26	69.80 (62.82–76.44)			
4–5 per day	47	76.44 (73.41–79.34)			
> 6 per day	21	76.18 (70.44–81.48)			
Assessment duration			2.40 (2)	0.301	
≤ 1 week	28	77.45 (71.71–82.56)			
> 1 but < 2 weeks	33	69.89 (65.12–74.46)			
≥ 2 weeks	65	76.10 (72.43–79.50)			
Clinical diagnosis			4.13 (1)	0.042	
Absent	111	76.02 (73.32–78.61)			
Present	15	69.80 (60.97–77.95)			
Substance of interest			6.30 (3)	0.098	
Tobacco	44	77.79 (73.76–82.64)			
Alcohol	44	76.36 (72.34–80.06)			
Marijuana	8	66.16 (57.88–74.02)			
Mixed	29	72.25 (66.26–77.87)			
Device			4.28 (4)	0.369	
PDA	63	74.37 (70.53–78.12)			
Internet	19	71.80 (63.68–79.26)			
Smartphone (own)	15	71.26 (64.07–77.95)			
Smartphone (loaned)	16	80.78 (76.10–85.14)			
IVR	9	76.36 (65.50–85.70)			
**Exploratory analyses**					
Event assessments			3.92 (1)	0.048	
Absent	68	72.79 (68.69–76.69)			
Present	58	77.61 (75.59–80.07)			
Training			0.67 (1)	0.413	
Absent	39	73.32 (68.32–78.17)			
Present	87	75.76 (72.52–78.77)			
Financial incentive			< 0.001	0.984	
Non‐structured	21	75.07 (68.04–81.04)			
Structured	76	75.24 (71.80–78.52)			
Treatment			0.92 (1)	0.337	
Yes	35	73.41 (68.32–78.53)			
No	91	75.84 (72.70–78.28)			
Motivation			1.37 (1)	0.242	
Absent	80	76.18 (72.87–79.43)			
Present	46	73.33 (68.89–77.54)			

CI = confidence interval; d.f. = degrees of freedom; IVR = interactive voice responding.

There were no significant differences in compliance as a result of prompt frequency or assessment duration; see Table 1. Individual assessment duration was not a significant predictor of compliance (coefficient = −0.04, SE = 0.01, 95% CI = –0.027, 0.194, *Z* = 0.33, *P* = 0.740). We report interactions between prompt frequency and total length of assessment in the Supporting information.

#### Measured substance use and clinical diagnosis

There was evidence that individuals with a clinical diagnosis of substance use disorder had reduced compliance compared to community‐based samples. There was no evidence that substance of assessment was associated with compliance rates.

#### Device administration

Three studies (2.3%) did not provide clear information on the device used to administer EMA prompts. There were no significant differences in compliance between the type of device used.

### Exploratory analyses

#### Treatment and motivation

There were no significant differences in compliance rates between studies when individuals were receiving treatment or not; furthermore, whether participants were motivated to reduce their substance use or not was not associated with compliance rates.

#### Event‐related assessments, training and reimbursement

There was no significant difference in compliance rates between studies that included event‐contingent assessments compared to those that did not. There was also no significant different in compliance rates between studies with and without explicit mention of participant training on the EMA protocol Similarly, there was no difference in compliance rates when structured financial incentives compared to non‐structured incentives were provided. 232 studies provided no information on participant reimbursement. Finally, there was no significant association between compliance rates and year of publication (coefficient = −0.007, SE = 0.004 (95% CI = –0.015–< 0.001) *Z* = 1.92, *P* = 0.055).

#### Trim‐and‐fill analysis

We conducted a trim‐and‐fill analysis 58 on our observed compliance rates to examine symmetry around the pooled‐estimate (see Supporting information, Fig. S2). The analyses identified 37 ‘missing’ effects which would have had lower rates of compliance (ranging from 28.21 to 55.45%). A revised estimate of overall compliance after inclusion of these studies was 70.81% (95% CI = 68.04–73.50%).

### Proportion of individuals not meeting minimum requirements for compliance

Thirty‐seven studies (*k* = 41 effect sizes) reported either excluding participants from the study due to inadequate compliance or the number of participants who did not meet minimum requirements for responding. On average, 6.20% (95% CI = 4.65–8.21%) of participants were excluded or did not meet the requirements across studies. We report exploratory analyses examining whether proposed moderators influenced exclusion/failure to meet minimum compliance rates in the Supporting information. To summarize, the number of prompts per day was negatively associated and length of assessment period was (weakly) positively associated with exclusion/failure to meet minimum requirements: participants were less likely to be excluded in studies with greater prompt frequency, and more likely to be excluded in studies of a longer duration. We urge caution in interpreting these findings due to the small number of valid effect sizes in the analyses.

## Discussion

The results from this meta‐analysis suggest that the pooled compliance rate for responding to signal‐contingent prompts in published EMA studies of substance use was outside the bounds of what is deemed acceptable 26, despite a large number of individual studies reporting acceptable levels. In line with previous observations 8 there was a large degree of heterogeneity in compliance rates. Our meta‐regressions identified that the presence of event related assessments increased compliance, and that participants with a diagnosis of substance use disorder had lower compliance rates.

The primary aim of this meta‐analysis was to provide a pooled estimate of compliance for EMA studies into substance use to provide a reference point for future research. The pooled compliance estimate was similar to those obtained from similar syntheses of EMA studies in other fields (children and adolescents 41), and higher than those in others (physical activity and dietary behaviours 40. However, it is worth considering that Liao *et al*. 41 also included paper‐and‐pencil assessments, which have been demonstrated to have substantially lower compliance than electronic assessments 59. Our analyses do not fully corroborate reports that individuals who use substances of abuse can adhere to intensive real‐time data collection in ambulatory settings 8, 60, as the pooled rate was lower than deemed acceptable and samples with a diagnosis of substance use disorder reported lower rates of compliance.

Our investigation into potential study‐level correlates of compliance rates limited evidence for our confirmatory hypotheses. We demonstrated no evidence that the number of prompt frequency influenced compliance rates, which contradicts recommendations by Burke *et al*. 3, who suggest that random prompts should be limited to a maximum of five per day in order to reduce participant burden. We also found no evidence that individual assessment duration, the device used to administer protocols or reimbursement influenced compliance, similar to previous findings 7, 32, 41. We demonstrated that the presence of event‐related assessments increased compliance, which may suggest an important role for participant engagement. Collectively, the present findings suggest that participants may comply with prompts in the real world, irrespective of assessment‐burden (within the limitations of our sample). However, care should be taken when interpreting these findings, as they refer to aggregate effects at the study level rather than at the individual participant level. As technology improves, future research should investigate how the design and acceptability of EMA software influences participant compliance.

Overall, the present study suggests that within the range limits reported in Table 1 the EMA assessment burden does not have an influence on the compliance rates. It is important for EMA researchers to take this message into account when designing EMA studies among substance users. Study protocols may have previously been informed by experience with EMA methods or pilot studies and focused on reduced participant burden. However, it may be possible to develop more intensive protocols to increase the amount of data collected without negatively influencing compliance. A potential caveat is that although participants may provide data, these data may not be of sufficient quality, and future research should investigate whether data quality is influenced by proposed moderators 61. An interesting example of this was demonstrated by Freedman *et al*. 62, who demonstrated reasonable compliance to a demanding telephone‐based EMA protocol in homeless crack cocaine addicts. However, qualitative analyses demonstrated addicts reported the protocol hard to tolerate, began to answer questions from memory and strategically answered questions (in this case reporting no craving) in an attempt to prevent follow‐up questions and shorten the testing session. This may be the case when reimbursement is structured around compliance rates.

There are limitations to our analyses. It is likely that the overall compliance rate was inflated, because some studies excluded individual participants who did not reach a specific rate of compliance (our Supporting information analyses demonstrated that approximately 6% of participants were excluded from studies due to poor compliance). There was considerable variability in these requirements, and it was often unclear if these decisions were made a priori. For example, some studies excluded participants who did not respond to at least 50% of signal‐contingent prompts 63, 64, showed less than 20% compliance 65 or excluded participants due to poor compliance without stating any criterion 66. Indeed, it is possible that studies aim for the acceptable rate of compliance (80%), as Fig. 2 demonstrates that the greatest frequency of compliance rates is between 80 and 85%. Furthermore, our funnel plots were asymmetrical, and a trim‐and‐fill analysis (see Supporting information) demonstrated a number of hypothetical missing effect sizes with lower rates of compliance. Secondly, we identified numerous studies that we could not include in our analyses due to an absence of compliance data reported. However, given the large number of effect sizes we identified, we still had considerable statistical power 67. 3Assuming detection of a small effect (*d* = 0.10) with an average of 154 participants per group and 126 effect sizes demonstrates that we have 1‐β = 0.99 (99% power), assuming high heterogeneity. Finally, it is possible that our coding for some of our moderators such as training and reimbursement was not sensitive, i.e. participants may have been reimbursed or trained, but this was not explicitly stated in the articles. For example, the amount of training provided to participants is difficult to quantify based on information provided in the Methods section. ‘Training’ might involve 10 minutes of training through familiarization or hours of intensive training with a researcher, and everything in between.

These limitations also point to a wider issue for EMA research regarding how missing data are reported and handled. Previous studies have attempted to provide guidelines for the reporting of EMA studies 26, 40, in particular the need for increased transparency when reporting compliance data. For example, minimum requirements might include a rationale for compliance decisions, the reporting of compliance by monitoring days and waves and reporting whether EMA compliance is related to procedural and individual factors. Future studies using EMA methodology should report data in line with these guidelines. In addition to improving reporting quality, future research should systematically examine variables which might improve compliance including ‘booster’ phone calls from researchers 68, interim compliance checks 69, coaching participants 27 and conveying the importance of good‐quality data 42. These strategies may be important, given that our exploratory analyses demonstrated that compliance rates were weakly negatively associated with year of study, which may suggest that EMA protocols may be becoming less acceptable for participants over time.

## Conclusions

To our knowledge, we present the first quantitative analyses of compliance to signal‐contingent assessments in EMA studies of substance use. Overall, we demonstrated compliance rates similar to those reported in other fields, although the average was slightly below the recommended rate of 80%. There was limited evidence that procedural variables or participant characteristics were robustly associated with compliance rates. A future challenge for the field is to identify methods to enhance compliance and also to improve reporting of missing data in line with published recommendations.

## Declarations of interest

None.

## Supporting information


**Table S1** Prisma checklist.
**Table S2** Studies included in the meta‐analyses and moderator coding.
**Figure S1** Forrest plot for overall compliance rate across all studies.
**Figure S2** Funnel plot with ‘filled’ data points from trim and fill analyses.
