# Supplementary material for: Compliance with ecological momentary assessment protocols in substance users: a meta‐analysis
Source: Addiction. 2018 Dec 21;114(4):609–19. doi: 10.1111/add.14503 (PMC6492133; doi:10.1111/add.14503)
Supplement: Supplementary file 1 — Table S1 Prisma checklist. Table S2 Studies included in the meta‐analyses and moderator coding. Figure S1 Forrest plot for overall compliance rate across all studies. Figure S2 Funnel plot with ‘filled’ data points from trim and fill analyses. [file ADD-114-609-s001.docx]

**Supplementary analyses:**

***Interaction between prompt frequency and total length of assessment period.***

We conducted a meta-regression including our coded factors of prompt frequency and total length of assessment period to examine the interaction between the two, however this model was not statistically significant Q(11) = 19.11, p = .059.

***Meta-regression on raw data for prompt frequency and length of assessment period.***

Conducting a meta-regression on raw prompt frequency and assessment duration demonstrated no significant relationship between number of prompts per day (Coefficient = -0.009, SE = 0.074, 95% CI = <-0.01, 0.02; z = 1.24, p = .216) or length of assessment period (Coefficient = <0.001, SE = <0.001, 95% CI = <-.001, >.001, Z = 1.25, p = .211), and the overall model did not meet statistical significance (Q(2) = 2.42, p = .298).

***Proportion of participants failing to meet minimum compliance or excluded due to poor compliance.***

We extracted data from 37 articles (contributing 41 effect sizes) which reported the number of participants failing to provide a minimum rate of compliance (e.g. 50%, Serre et al, 2012) or specifically stating the number of participants who were excluded based on poor compliance (e.g. *‘students who did not complete at least 15 diary entries (…) were excluded’*, Armeli et al, 2014).^[[1]](#footnote-1)^ We used the same formula for compliance rates to compute the effect size for proportion of individuals not meeting requirements. However as there is no variance within studies as to the number of participants not meeting requirements standard error was calculated as √(p(1-p)/n), where p is the proportion and n is the number of participants in the study.

Overall the percentage of participants who did not meet minimum requirements / were excluded in studies was 6.20% (95% CI 4.65% – 8.21%). Meta-regressions demonstrated that there was a significant negative association between prompt frequency and percentage of participants who did not meet compliance requirements (Coefficient = -0.029 (95% CI -0.042 - -0.015); z = -4.30, p < .001). There was also a weak positive association with length of assessment period (Coefficient = .001 (95% CI <-.001 - .001); Z = 1.75, p = .081). These findings should be interpreted with caution due to a small number of eligible effect sizes.

**Supplementary Table 1: PRISMA CHECKLIST**

| **Section/topic** | | **#** | **Checklist item** | **Reported on page #** | |
| --- | --- | --- | --- | --- | --- |
| **TITLE** | | | |  | |
| Title | | 1 | Identify the report as a systematic review, meta-analysis, or both. | 1 | |
| **ABSTRACT** | | | |  | |
| Structured summary | | 2 | Provide a structured summary including, as applicable: background; objectives; data sources; study eligibility criteria, participants, and interventions; study appraisal and synthesis methods; results; limitations; conclusions and implications of key findings; systematic review registration number. | 2 | |
| **INTRODUCTION** | | | |  | |
| Rationale | | 3 | Describe the rationale for the review in the context of what is already known. | 3-6 | |
| Objectives | | 4 | Provide an explicit statement of questions being addressed with reference to participants, interventions, comparisons, outcomes, and study design (PICOS). | 6-7 | |
| **METHODS** | | | |  | |
| Protocol and registration | | 5 | Indicate if a review protocol exists, if and where it can be accessed (e.g., Web address), and, if available, provide registration information including registration number. | 7 | |
| Eligibility criteria | | 6 | Specify study characteristics (e.g., PICOS, length of follow-up) and report characteristics (e.g., years considered, language, publication status) used as criteria for eligibility, giving rationale. | 8 | |
| Information sources | | 7 | Describe all information sources (e.g., databases with dates of coverage, contact with study authors to identify additional studies) in the search and date last searched. | 7 | |
| Search | | 8 | Present full electronic search strategy for at least one database, including any limits used, such that it could be repeated. | 7 | |
| Study selection | | 9 | State the process for selecting studies (i.e., screening, eligibility, included in systematic review, and, if applicable, included in the meta-analysis). | 8-9 | |
| Data collection process | | 10 | Describe method of data extraction from reports (e.g., piloted forms, independently, in duplicate) and any processes for obtaining and confirming data from investigators. | 8-9 | |
| Data items | | 11 | List and define all variables for which data were sought (e.g., PICOS, funding sources) and any assumptions and simplifications made. | 8-9 | |
| Risk of bias in individual studies | | 12 | Describe methods used for assessing risk of bias of individual studies (including specification of whether this was done at the study or outcome level), and how this information is to be used in any data synthesis. | N/A | |
| Summary measures | | 13 | State the principal summary measures (e.g., risk ratio, difference in means). | 11 | |
| Synthesis of results | | 14 | Describe the methods of handling data and combining results of studies, if done, including measures of consistency (e.g., I^2^) for each meta-analysis. | 10-11 | |
| Risk of bias across studies | | 15 | Specify any assessment of risk of bias that may affect the cumulative evidence (e.g., publication bias, selective reporting within studies). | 15 | |
| Additional analyses | | 16 | Describe methods of additional analyses (e.g., sensitivity or subgroup analyses, meta-regression), if done, indicating which were pre-specified. | 14-15 | |
| **RESULTS** | | | |  |  |
| Study selection | | 17 | Give numbers of studies screened, assessed for eligibility, and included in the review, with reasons for exclusions at each stage, ideally with a flow diagram. | 12 |  |
| Study characteristics | | 18 | For each study, present characteristics for which data were extracted (e.g., study size, PICOS, follow-up period) and provide the citations. | supplementary |  |
| Risk of bias within studies | | 19 | Present data on risk of bias of each study and, if available, any outcome level assessment (see item 12). | N/A |  |
| Results of individual studies | | 20 | For all outcomes considered (benefits or harms), present, for each study: (a) simple summary data for each intervention group (b) effect estimates and confidence intervals, ideally with a forest plot. | 12-15, figure 2 and supplementary |  |
| Synthesis of results | | 21 | Present results of each meta-analysis done, including confidence intervals and measures of consistency. | Table 2 |  |
| Risk of bias across studies | | 22 | Present results of any assessment of risk of bias across studies (see Item 15). | N/A |  |
| Additional analysis | | 23 | Give results of additional analyses, if done (e.g., sensitivity or subgroup analyses, meta-regression [see Item 16]). | 13 |  |
| **DISCUSSION** | | | |  |  |
| Summary of evidence | | 24 | Summarize the main findings including the strength of evidence for each main outcome; consider their relevance to key groups (e.g., healthcare providers, users, and policy makers). | 16-19 |  |
| Limitations | | 25 | Discuss limitations at study and outcome level (e.g., risk of bias), and at review-level (e.g., incomplete retrieval of identified research, reporting bias). | 18 |  |
| Conclusions | | 26 | Provide a general interpretation of the results in the context of other evidence, and implications for future research. | 19 |  |
| **FUNDING** | | | |  |  |
| Funding | 27 | | Describe sources of funding for the systematic review and other support (e.g., supply of data); role of funders for the systematic review. | 19 |  |

**Supplementary Table 2: Studies included in the meta-analyses and moderator coding.**

| **RECORD** | **AUTHORS** | **YEAR** | **DELIVERY** | **DEPENDENT** | **SUBSTANCE** | **TREATMENT** | **QUIT?** | **PROMPTS_PER_DAY** | **NUMBER_OF_DAYS** | **ASSESSMENT_MINS** | **REIMBURSED** | **EVENT** | **TRAINIING** |
| --- | --- | --- | --- | --- | --- | --- | --- | --- | --- | --- | --- | --- | --- |
| 1 | Aigner et al | 2016 | 1 | 0 | 2 | 1 | 1 | 1 | 14 |  |  | 0 | 0 |
| 2 | Aldridge-Gerry | 2011 | 2 | 0 | 1 | 0 | 0 | 1 | 5 | 5.00 | 1 | 0 | 0 |
| 3 | Ansell et al | 2015 | 3 | 0 | 5 | 0 | 0 | 1 | 14 |  | 2 | 0 | 1 |
| 4 | Armeli et al | 2014 | 2 | 0 | 1 | 0 | 0 | 1 | 30 |  | 1 | 0 | 0 |
| 5 | Armeli et al | 2003 | 1 | 0 | 1 | 0 | 0 | 3 | 30 |  |  | 0 | 0 |
| 6 | Battista et al | 2015 | 1 | 0 | 1 | 0 | 0 | 6 | 22 |  | 2 | 0 | 1 |
| 7 | Berkman et al | 2011 | 3 | 0 | 2 | 1 | 1 | 8 | 21 |  | 2 | 0 | 0 |
| 8 | Berli et al | 2015 | 4 | 0 | 2 | 0 | 1 | 1 | 32 |  |  | 0 | 0 |
| 9 | Bernhardt et al | 2007 | 1 | 0 | 1 | 0 | 0 | 1 | 14 |  |  | 0 | 0 |
| 10 | Bernhardt et al | 2009 | 4 | 0 | 1 | 0 | 0 | 1 | 30 |  |  | 0 | 0 |
| 11 | Black et al | 2013 | 1 | 0 | 3 | 0 | 0 | 4 | 15 | 2.50 | 2 | 1 | 0 |
| 12 | Bold et al | 2016 | 1 | 0 | 2 | 1 | 1 | 4 | 21 | 5.00 |  | 0 | 1 |
| 13 | Boytnton et al | 2014 | 2 | 0 | 1 | 0 | 0 | 1 | 14 | 5.00 | 2 | 0 | 0 |
| 14 | Bravo | 2017 | 2 | 0 | 3 | 0 | 0 | 1 | 12 | 10.00 | 2 | 0 | 0 |
| 15 | Brodbeck | 2014 | 3 | 0 | 2 | 0 | 1 | 3 | 28 |  | 1 | 0 | 0 |
| 16 | Bruckner et al | 2016 | 1 | 0 | 2 | 0 | 1 | 3 | 14 |  |  | 0 | 1 |
| 17 | Bruckner et al | 2015 | 1 | 0 | 3 | 0 | 0 | 6 | 14 |  | 2 | 1 | 1 |
| 18 | Bruckner et al | 2013 | 1 | 0 | 3 | 0 | 1 | 6 | 14 |  | 2 | 1 | 1 |
| 19 | Buckner et al | 2012 | 1 | 0 | 3 | 0 | 0 | 6 | 14 |  | 1 | 1 | 1 |
| 20 | Businelle et al | 2014 | 4 | 0 | 2 | 1 | 1 | 4 | 6 |  | 2 | 1 | 1 |
| 21 | Businelle et al | 2016 | 4 | 1 | 2 | 1 | 1 | 4 | 21 | 5.00 | 2 | 1 | 1 |
| 22 | Cerrada et al | 2016 | 3 | 0 | 2 | 0 | 0 | 5 | 7 |  |  | 1 | 0 |
| 23 | Collins et al | 1998 | 1 | 0 | 1 | 1 | 1 | 6 | 56 |  | 1 | 1 | 1 |
| 24 | Comulanda et al | 2015 | 4 | 1 | 5 | 1 | 1 | 1 | 30 |  | 2 | 1 | 1 |
| 25 | Dennis et al | 2016 | 1 | 0 | 2 | 1 | 1 | 6 | 7 |  | 2 | 1 | 0 |
| 26 | Denns et al | 2015 | 4 | 1 | 5 | 1 | 1 | 6 | 7 | 2.50 | 2 | 0 | 1 |
| 27 | Dvorak et al | 2014 | 1 | 0 | 1 | 0 | 0 | 8 | 21 |  | 1 | 0 | 1 |
| 28 | Dvoral et al | 2014 | 1 | 0 | 1 | 0 | 0 | 9 | 21 |  | 2 | 1 | 1 |
| 29 | Dworkin | 2017 | 2 | 0 | 3 | 0 | 0 | 2 | 28 | 7.50 | 1 | 0 | 1 |
| 30 | Ehernberg et al | 2016 | 2 | 0 | 1 | 0 | 0 | 1 | 30 |  | 1 | 0 | 0 |
| 31 | Epstein et al | 2010 | 1 | 1 | 5 | 1 | 1 | 3 | 175 |  |  | 1 | 0 |
| 32 | Etcheverry et al | 2016 | 1 | 0 | 2 | 0 | 1 | 4 | 8 | 3.27 | 2 | 1 | 1 |
| 33 | Gaher et al | 2014 | 1 | 0 | 1 | 0 | 0 | 8 | 14 | 2.00 | 2 | 0 | 1 |
| 34 | Gass et al | 2012 | 1 | 0 | 2 | 1 | 1 | 2 | 30 |  | 2 | 0 | 1 |
| 35 | Groefsema et al | 2016 | 3 | 0 | 1 | 0 | 0 | 6 | 15 |  | 2 | 0 | 0 |
| 36 | Gwaltney et al | 2008 | 1 | 0 | 2 | 0 | 1 | 4 | 21 |  | 1 | 1 | 1 |
| 37 | Hederker et al | 2009 | 1 | 0 | 2 | 0 | 0 | 5 | 7 |  |  | 1 | 0 |
| 38 | Herbert et al | 2017 | 3 | 0 | 2 | 0 | 0 | 1 | 28 |  | 2 | 1 | 0 |
| 39 | Hoeppner et al | 2014 | 1 | 0 | 2 | 0 | 1 | 8 | 21 |  | 2 | 1 | 1 |
| 40 | Hruska et al | 2017 | 1 | 0 | 1 | 0 | 0 | 3 | 7 |  | 2 | 0 | 1 |
| 41 | Hufford et al | 2002 | 1 | 0 | 1 | 0 | 0 | 5 | 14 |  | 1 | 1 | 1 |
| 42 | Huh et al | 2014 | 3 | 0 | 2 | 0 | 0 | 5 | 7 |  |  | 1 | 1 |
| 43 | Johnson et al | 2009 | 1 | 1 | 5 | 1 | 1 | 5 | 7 |  | 2 | 0 | 1 |
| 44 | Kennedy et al | 2013 | 1 | 1 | 5 | 1 | 1 | 4 | 175 |  |  | 1 | 0 |
| 45 | Kiene et al | 2009 | 2 | 0 | 1 | 0 | 0 | 1 | 30 |  | 2 | 0 | 0 |
| 46 | Kirchner et al | 2013 | 4 | 0 | 2 | 0 | 1 | 3 | 30 |  |  | 0 | 0 |
| 47 | Kuntsche et al | 2013 | 3 | 0 | 1 | 0 | 0 | 2 | 30 | 1.00 | 2 | 0 | 0 |
| 48 | Lam et al | 2014 | 1 | 0 | 5 | 1 | 1 | 4 | 7 | 3.00 | 2 | 1 | 1 |
| 49 | Lane et al | 2016 | 1 | 0 | 1 | 0 | 0 | 6 | 21 |  | 2 | 1 | 0 |
| 50 | Langdone et al | 2016 | 1 | 0 | 2 | 1 | 1 | 3 | 14 |  |  | 0 | 0 |
| 51 | MacClean et al | 2017 |  | 0 | 2 | 0 | 1 | 7 | 8 |  |  | 1 | 0 |
| 52 | Marhe et al | 2013 | 1 | 1 | 5 | 1 | 1 | 4 | 7 |  | 1 | 1 | 1 |
| 53 | Martino et al | 2016 | 4 | 0 | 1 | 0 | 0 | 3 | 14 |  | 2 | 1 | 1 |
| 54 | McCarthy et al | 2006 | 1 | 0 | 2 | 1 | 1 | 4 | 42 | 2.30 | 2 | 0 | 1 |
| 55 | Messieh | 2011 | 1 | 0 | 5 | 0 | 0 | 5 | 7 | 1.50 | 1 | 0 | 1 |
| 56 | Mohr et al | 2015 | 1 | 0 | 1 | 0 | 0 | 3 | 30 |  | 2 | 0 | 1 |
| 57 | Monk et al | 2014 | 3 | 0 | 1 | 0 | 0 | 5 | 7 |  |  | 0 | 1 |
| 58 | Moore et al | 2014 | 1 | 1 | 5 | 1 | 1 | 3 | 120 |  | 2 | 0 | 1 |
| 59 | Moore et al | 2011 | 1 | 0 | 1 | 0 | 0 | 1 | 60 |  | 2 | 0 | 1 |
| 60 | Muraven et al | 2005 | 1 | 0 | 1 | 0 | 0 | 4 | 14 |  |  | 1 | 1 |
| 61 | Mustanski | 2008 | 2 | 0 | 1 | 0 | 0 | 1 | 30 | 5.00 | 2 | 0 | 0 |
| 62 | O'Grady et al | 2011 | 2 | 0 | 1 | 0 | 0 | 1 | 30 | 5.00 |  | 0 | 1 |
| 63 | O'Grady et al | 2012 | 1 | 0 | 1 | 0 | 0 | 1 | 21 |  | 2 | 0 | 1 |
| 64 | O'Grady et al | 2012 | 2 | 0 | 1 | 0 | 0 | 1 | 21 |  | 2 | 0 | 1 |
| 65 | Otsuki | 2009 | 1 | 0 | 2 | 0 | 0 | 5 | 7 | 2.00 | 1 | 0 | 1 |
| 66 | Patrick and Lee | 2010 | 2 | 0 | 1 | 0 | 0 | 1 | 29 | 7.50 | 2 | 0 | 0 |
| 67 | Peacock et al | 2015 | 4 | 0 | 1 | 0 | 0 | 4 | 10 |  | 1 | 1 | 1 |
| 68 | Phillips et al | 2015 | 3 | 0 | 3 | 0 | 0 | 3 | 14 |  | 1 | 0 | 1 |
| 69 | Piasecki et al | 2011 | 1 | 0 | 5 | 0 | 0 | 5 | 21 |  | 2 | 1 | 1 |
| 70 | Piasecki et al | 2014 | 1 | 0 | 2 | 0 | 0 | 5 | 7 | 1.50 |  | 1 | 0 |
| 71 | Piper et al | 2011 | 1 | 0 | 2 | 1 | 1 | 4 | 20 |  |  | 0 | 1 |
| 72 | Polak and Conner | 2012 | 2 | 0 | 1 | 0 | 0 | 1 | 21 | 5.00 |  | 0 | 1 |
| 73 | Preston et al | 2017 | 4 | 1 | 5 | 1 | 1 | 3 | 112 |  | 2 | 1 | 1 |
| 74 | Ramirez and Miranda | 2014 | 1 | 0 | 1 | 0 | 0 | 3 | 7 |  |  | 1 | 1 |
| 75 | Rendina et al | 2016 | 2 | 0 | 5 | 0 | 0 | 1 | 30 | 4.00 | 2 | 0 | 0 |
| 76 | Reynolds et al | 2015 | 4 | 0 | 5 | 0 | 0 | 1 | 84 |  | 2 | 0 | 1 |
| 77 | Roberts et al | 2017 |  | 0 | 2 | 0 | 0 | 3 | 10 | 1.50 | 2 | 0 | 1 |
| 78 | Rowan et al | 2007 | 1 | 0 | 2 | 1 | 1 | 4 | 7 | 3.00 | 2 | 1 | 1 |
| 79 | Ruscio et al | 2016 | 1 | 0 | 2 | 1 | 0 | 4 | 14 |  | 2 | 1 | 1 |
| 80 | Scharf et al | 2013 | 4 | 0 | 5 | 0 | 0 | 2 | 14 |  | 1 | 1 | 1 |
| 81 | Schuster et al | 2016 | 1 | 0 | 5 | 0 | 0 | 5 | 7 | 3.80 | 2 | 1 | 1 |
| 82 | Schuz et al | 2016 | 4 | 0 | 2 | 1 | 1 | 5 | 16 |  | 1 | 1 | 1 |
| 83 | Schuz et al | 2016 | 4 | 0 | 2 | 1 | 1 | 5 | 28 |  |  | 1 | 0 |
| 84 | Serre et al | 2012 | 1 | 1 | 5 | 1 | 1 | 4 | 14 | 4.10 | 2 | 0 | 1 |
| 85 | Setodki | 2013 | 1 | 0 | 2 | 0 | 0 | 3 | 21 |  | 2 | 1 | 1 |
| 86 | Shiffman et al | 2014 | 1 | 0 | 2 | 0 | 0 | 4 | 21 |  |  | 1 | 1 |
| 87 | Shiffman et al | 2002 | 1 | 0 | 2 | 1 | 1 | 5 | 7 |  | 1 | 1 | 1 |
| 88 | Shorey et al | 2014 | 2 | 0 | 5 | 0 | 0 | 1 | 90 |  | 2 | 0 | 0 |
| 89 | Shorey et al | 2014 | 2 | 0 | 5 | 0 | 0 | 1 | 90 | 5.00 | 2 | 0 | 0 |
| 90 | Shrier et al | 2012 | 1 | 0 | 3 | 0 | 0 | 5 | 14 | 2.00 | 2 | 0 | 1 |
| 91 | Shuz et al | 2014 | 4 | 0 | 2 | 0 | 1 | 5 | 6 |  | 1 | 1 | 1 |
| 92 | Simons et al | 2010 | 1 | 0 | 1 | 0 | 0 | 8 | 21 | 1.50 | 2 | 1 | 1 |
| 93 | Simons et al | 2014 | 1 | 0 | 1 | 0 | 0 | 7 | 49 | 2.00 | 2 | 1 | 1 |
| 94 | Smiley et al | 2017 | 3 | 0 | 5 | 0 | 0 | 3 | 14 |  | 2 | 0 | 1 |
| 95 | Sokolovsky et al | 2014 | 1 | 0 | 2 | 0 | 0 | 6 | 7 |  |  | 0 | 1 |
| 96 | Stevens et al | 2017 | 2 | 0 | 1 | 0 | 0 | 1 | 10 |  |  | 0 | 0 |
| 97 | Swenden et al | 2000 | 1 | 0 | 1 | 0 | 0 | 3 | 30 |  |  | 1 | 1 |
| 98 | Thrul | 2015 | 3 | 0 | 2 | 0 | 1 | 5 | 3 | 2.00 | 2 | 1 | 1 |
| 99 | Tidey et al | 2008 | 1 | 0 | 1 | 0 | 0 | 5 | 21 |  | 2 | 1 | 1 |
| 100 | Tracy DeHart | 2009 | 2 | 0 | 1 | 0 | 0 | 1 | 30 |  | 2 | 0 | 0 |
| 101 | Trull et al | 2016 | 1 | 0 | 5 | 0 | 0 | 6 | 28 |  | 2 | 0 | 0 |
| 102 | Turner et al | 2017 | 3 | 0 | 5 | 1 | 1 | 3 | 60 |  | 2 | 0 | 1 |
| 103 | Usdan et al | 2004 | 2 | 0 | 1 | 0 | 0 | 1 | 35 |  | 2 | 0 | 1 |
| 104 | Van Zundert et al | 2010 | 2 | 0 | 2 | 0 | 1 | 3 | 28 | 3.00 |  | 0 | 0 |
| 105 | Vinci et al | 2017 | 1 | 0 | 2 | 1 | 1 | 4 | 32 | 2.50 |  | 1 | 0 |
| 106 | Warthen & Tiffany | 2009 | 1 | 0 | 2 | 0 | 0 | 4 | 8 |  | 2 | 1 | 1 |
| 107 | Waters & Li | 2008 | 1 | 0 | 2 | 0 | 0 | 4 | 7 |  | 2 | 1 | 1 |
| 108 | Waters & Miller | 2010 | 1 | 0 | 2 | 0 | 0 | 4 | 7 |  | 2 | 1 | 0 |
| 109 | Waters et al | 2012 | 1 | 1 | 5 | 1 | 1 | 4 | 7 | 7.10 | 2 | 1 | 1 |
| 110 | Weinstein et al | 2006 | 1 | 0 | 2 | 0 | 0 | 5 | 7 |  | 1 | 1 | 1 |
| 111 | Wetter et al | 2011 | 1 | 0 | 2 | 1 | 1 | 4 | 7 |  | 2 | 1 | 1 |
| 112 | Witkiewitz et al | 2014 | 3 | 0 | 5 | 1 | 0 | 3 | 14 | 4.00 | 2 | 1 | 1 |
| 113 | Witkiewitz et al | 2012 |  | 0 | 5 | 0 | 0 | 3 | 21 |  | 2 | 1 | 1 |
| 114 | Wray et al | 2011 | 1 | 0 | 2 | 0 | 0 | 4 | 8 | 3.00 | 2 | 0 | 1 |
| 115 | Wray et al | 2015 | 3 | 0 | 2 | 0 | 0 | 4 | 14 |  | 2 | 0 | 1 |
| 116 | Wray et al | 2016 | 4 | 0 | 5 | 0 | 0 | 6 | 30 | 1.50 | 2 | 1 | 1 |
| 117 | Yang et al | 2015 | 4 | 0 | 5 | 0 | 1 | 3 | 28 | 1.15 | 2 | 1 | 1 |
| 118 | Cohen et al | 2014 | 5 | 0 | 1 | 0 | 0 | 1 | 14 | 6.00 | 2 | 0 | 1 |
| 119 | Collins et al | 2003 | 5 | 0 | 1 | 0 | 0 | 4 | 14 | 10.00 | 1 | 1 | 1 |
| 120 | Helzer et al | 2006 | 5 | 0 | 1 | 0 | 0 | 1 | 730 | 2.00 | 2 | 0 | 1 |
| 121 | Holz et al | 2012 | 5 | 1 | 5 | 1 | 1 | 5 | 28 |  | 2 | 0 | 1 |
| 122 | Neupert et al | 2017 | 5 | 1 | 5 | 1 | 1 | 1 | 14 | 5.00 | 2 | 0 | 1 |
| 123 | Possemato et al | 2015 | 5 | 0 | 1 | 0 | 0 | 4 | 28 |  | 2 | 0 | 1 |
| 124 | Barta et al | 2008 | 5 | 0 | 1 | 0 | 0 | 1 | 35 | 10.00 | 2 | 0 | 1 |
| 125 | Freeman et al | 2006 | 5 | 1 | 4 | 1 | 1 | 8 | 14 |  | 2 | 0 | 1 |
| 126 | Simpson et al | 2005 | 5 | 1 | 1 | 1 | 1 | 1 | 28 | 3.80 | 2 | 0 | 1 |

***Labels: Delivery= device used. Dependent = clinical diagnosis of SUD. Substance = main substance of interest. Event = event related assessment present. Training = training present. Prompts per day = the number of EMA prompts received each day. Assessment mins = length of individual EMA assessment in minutes.***

***Coding: Delivery: 1 (PDA), 2 (INTERNET), 3 (PHONE_OWN), 4 (PHONE_LOAN) 5 (IVF); Substance: 1 (ALCOHOL), 2 (NICOTINE), 3 (MARIJUANA), 4 (ILLICIT), 5 (MIXED). Binary variables (0 = ABSENT, 1 = PRESENT). Reimbursement (1 = fixed, 2 = structured).***

***Supplementary Figure 1: Forrest plot for overall compliance rate across all studies. ^[[2]](#footnote-2)^
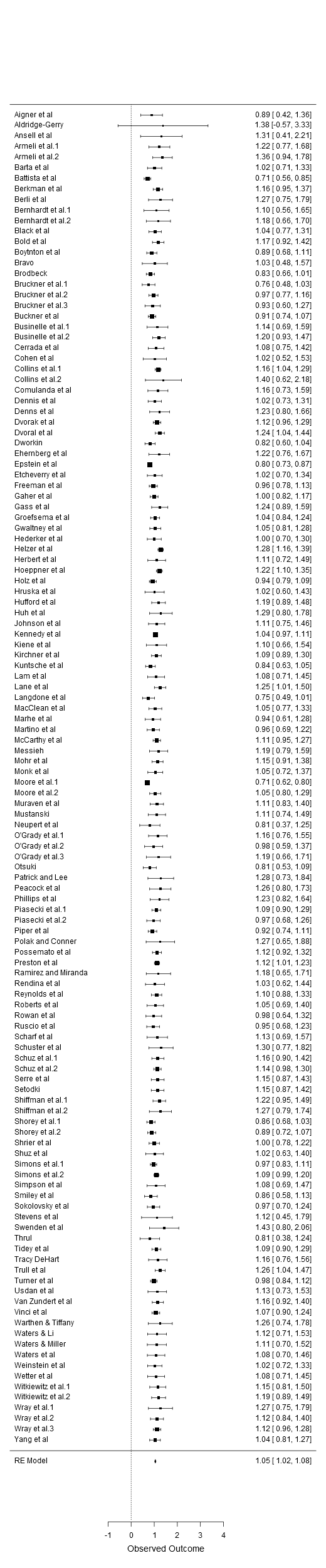
***

***Figure caption: Observed outcome relates to the (square-root of the arcsine) transformed compliance rates. 95% Confidence intervals of this outcome are in brackets. RE model = Random Effects Model***

***Supplementary Figure 2: Funnel plot with ‘filled’ data points from trim and fill analyses.*** ^[[3]](#footnote-3)^


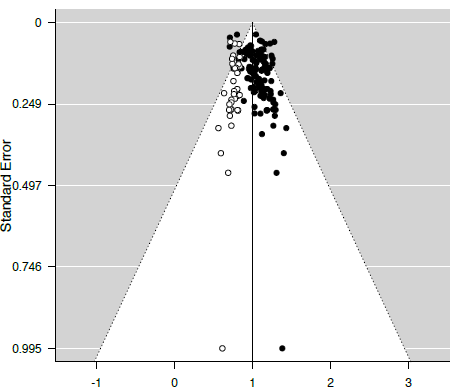


1. Some studies (e.g. Aigner et al, 2016; Businelle et al 2016) reported excluding participants who did not provide *any* EMA data. We did not include these as these would not give any indication that the experience of the EMA protocol would influence compliance. [↑](#footnote-ref-1)
2. PDF version available on OSF (https://osf.io/bwmup/) [↑](#footnote-ref-2)
3. Note, the study with the large SE is Aldridge-Gerry (2011) who had a small number of total prompts per protocol (1 per-day for 5 days). [↑](#footnote-ref-3)
